# Efficacy and safety of external therapy of TCM for primary dysmenorrhea: A protocol for systematic review and meta-analysis

**DOI:** 10.1097/MD.0000000000029155

**Published:** 2022-05-13

**Authors:** Haiyu Zhu, Hongyi Guan, Tingwei Ding, Yunpeng Bi, Yue Zhuo, Yuanyuan Chen, Tie Li, Zhihong Wang

**Affiliations:** Department of Acupuncture and Tuina, Changchun University of Chinese Medicine, Changchun, China.

**Keywords:** acupuncture, meta-analysis, primary dysmenorrhea, protocol, systematic review, Tuina

## Abstract

**Background:**

Primary dysmenorrhea (PD) is a painful disease that severely affects the daily lives of young women. External therapy using traditional Chinese medicine (TCM) is widely used in the clinical treatment of this disease and has achieved a good effect. There are many kinds of TCM external therapies, but the effectiveness, safety, and advantages of various methods have not been confirmed by high-quality meta-analyses. The purpose of this study was to evaluate the efficacy and safety of representative TCM external therapies in patients with PD.

**Methods:**

Published randomized controlled trials that assessed the efficacy of these interventions in patients with PD were included. We searched the following electronic databases: PubMed, Cochrane Library, Embase, Web of Science, China Science and Technology Journal, China National Knowledge Infrastructure, Wanfang, and Chinese Biomedical Literature Database and other sources. The main outcomes included menstrual pain intensity and the clinical effectiveness rate. The secondary outcomes were the quality of life and safety. The meta-analysis will be performed using the Review Manager version 5.3 software.

**Results:**

We will provide more practical results investigating the efficacy of external TCM therapy with high clinical frequency for PD patients in several respects, including the improvement of menstrual pain intensity, quality of life, and safety.

**Conclusion:**

This review systematically reviews the effectiveness and safety of common TCM external therapies for PD and provides clinicians with the best treatment options.

**Ethics and dissemination:**

This systematic review aimed to evaluate the efficacy and safety of external TCM therapy for PD. Since all data included were published, the systematic review did not require ethical approval.

**Registration number:**

CRD42021297078.

## 1 Introduction

Primary dysmenorrhea (PD) is to point to below the circumstance that does not have pelvic cavity pathology change, the next abdomen ache that happens in menstruation or early menstruation, companion has the radiative ache of recurrent spasmodic sex and lumbosacral ministry.^[1]^ Its first signs usually appear approximately 6 months after menarche.^[2]^ The pain usually lasts from 8 to 72 hours and is usually accompanied by nausea, vomiting, headache, and hyperthermia.^[3]^ It affects 50% to 90% of women, and they describe pain as moderate to severe. More than half of the patients said that they restricted their daily work and study, and 17 percent said they were out of school or unemployed.^[1]^ Although the incidence is high, it is often overlooked. One reason is that some women think menstrual cycle pain is a normal phenomenon, and the other reason is inaccurate diagnosis or inappropriate treatment, which causes them to experience a lot of pain.^[4]^

PD is caused by excessive contraction of the uterine muscle layer, leading to uterine muscle ischemia and hypoxia and resulting in pain.^[5]^ The causes of this are still being investigated. However, excessive contraction of the uterine muscle layer caused by the increase in menstrual prostaglandin levels has become increasingly stable.^[6–8]^ Studies have shown that patients with PD have higher levels of prostaglandins released when their endometrium falls off than healthy women.^[3]^ From the point of view of traditional Chinese medicine (TCM), the cause of PD is mainly dysfunction of the viscera, qi, and blood block and so on.

Currently, the treatment of PD is based on pain relief, including drug and external therapies. Drug therapy involves the administration of nonsteroidal anti-inflammatory drugs and hormones.^[9–12]^ These drugs have achieved good clinical efficacy; however, they have certain side effects, such as liver and kidney damage and gastric mucosa irritation, and are not suitable for long-term use.^[13]^ The external treatment of TCM includes acupuncture, massage, acupoint application, and heat therapy, which has a very good effect on pain relief and is safer.^[14]^

According to ancient records of TCM, acupuncture, massage, acupoint sticking, and other therapeutic methods can promote blood circulation and regulate uterine function.^[15,16]^ Previous randomized controlled trials (RCTs) have confirmed the effectiveness of external TCM therapy in the treatment of PD. However, to date, there has been no systematic review or rigorously designed meta-analysis evaluating TCM external therapy for treating PD. Therefore, our research team is planning a systematic review and meta-analysis through the typical external therapy of TCM treatment, including acupuncture massage and acupoint sticking, and investigating the treatment of PD clinical efficacy and safety of clinical medicine in future research will provide true and reliable evidence.

## 2 Methods

### 2.1 Study registration

This systematic review protocol was registered with the PROSPERO Network (No. CRD42021297078). This protocol adhered to the preferred reporting items for systematic reviews and meta-analysis protocols in 2015.^[17]^

### 2.2 Inclusion criteria

#### 2.2.1 Types of studies

All RCTs that stated the “randomization” phrase will be included, regardless of allocation concealment or use of blinding, and published or unpublished RCTs without language restriction. Studies should be available in full and peer-reviewed.

#### 2.2.2 Types of participants

This study will employ the diagnostic standards of the Clinical Guideline for PD by the Society of Obstetricians and Gynecologists of Canada.^[2]^ Does not include chronic pelvic inflammation, gynecological malignant tumors, endometriosis, secondary dysmenorrhea, or other gynecological diseases. No sex, race, nationality, or comorbidity was limited.

#### 2.2.3 Interventions and comparators

The experimental group will include all types of external TCM treatments for PD, such as acupuncture, Tuina, moxibustion, acupoint sticking, and auricular acupuncture. The control group was treated with sham acupuncture, placebo, or conventional medicine, or no treatment. When studies combine one of the external treatments of TCM with other active therapies, both experimental and control groups are required to use the same active therapy.

#### 2.2.4 Outcome

The primary outcomes include:

- Menstrual pain intensity: Pain intensity was indicated by a visual analog scale or a numerical rating scale.
- Clinical effectiveness rate: an overall reduction in symptoms (a reduction in menstruation-related symptoms that occur only during the intervention or as a result of the intervention, including dysmenorrhea).

The secondary outcomes include:

- Quality of life was measured using valid questionnaires.
- Adverse events.

### 2.3 Search methods for the identification of studies

The following electronic databases will be searched from the respective dates of database inception to October 10, 2021: Cochrane Library, PubMed, Web of Science, EMBASE, China Science and Technology Journal, China National Knowledge Infrastructure, Wanfang, Chinese Biomedical Literature Database, and other sources. All published English and Chinese RCTs were included.

The retrieval mode used will be a combination of free words and medical subject headings terms, including “dysmenorrhea”, “primary dysmenorrhea”, “menstrual pain”, “painful menstruation”, “period pain”, “menstrual disorder”, “pelvic pain”, “meralgia”, “moxibustion”, “suspended moxibustion”, “mild moxibustion”, “acupuncture”, “acupuncture therapy”, “electroacupuncture”, “auriculotherapy”, “acupoint”, “acupoint sticking therapy”, “acupoint catgut embedding”, “Tuina”, “Chinese Tuina”, “massage”, “massage therapy”, “Chinese massage”, “Manipulation”. The search strategy takes PubMed as an example, as shown in Table 1.

**Table 1:** Search strategy for the PubMed database.

Number	Terms
#1	Dysmenorrhea (all field)
#2	Primary dysmenorrhea (all field)
#3	Menstrual pain (all field)
#4	Painful menstruation (all field)
#5	Period pain (all field)
#6	Painful period (all field)
#7	Menstrual disorder (all field)
#8	#1 OR #2-7
#9	Acupuncture (all field)
#10	Needling (all field)
#11	Acupoint (all field)
#12	Scalp acupuncture (all field)
#13	Ear acupuncture (all field)
#14	Massage (all field)
#15	Acupoints (all field)
#16	Tuina (all field)
#17	Manipulation (all field)
#18	Auricular acupuncture (all field)
#19	Electroacupuncture (all field)
#20	Acupoint sticking (all field)
#21	Catgut embedding (all field)
#22	#9 OR #10-21
#23	Randomized controlled trial (all field)
#24	Controlled clinical trial (all field)
#25	Randomly (all field)
#26	Randomized (all field)
#27	Random allocation (all field)
#28	Placebo (all field)
#29	Double-blind method (all field)
#30	Single-blind method (all field)
#31	Trials (all field)
#32	#23 OR #24-31
#33	#8 and #22 and #32

### 2.4 Exclusion criteria

The exclusion criteria contain the following items:

- Patients with organic diseases or pregnant women.
- Non-RCT reviews, animal experiments, case reports, expert experience, and conference articles.
- Incomplete data or information.
- Repeatedly checked or published literature.

### 2.5 Data collection and analysis

#### 2.5.1 Study selection

**Figure 1:**
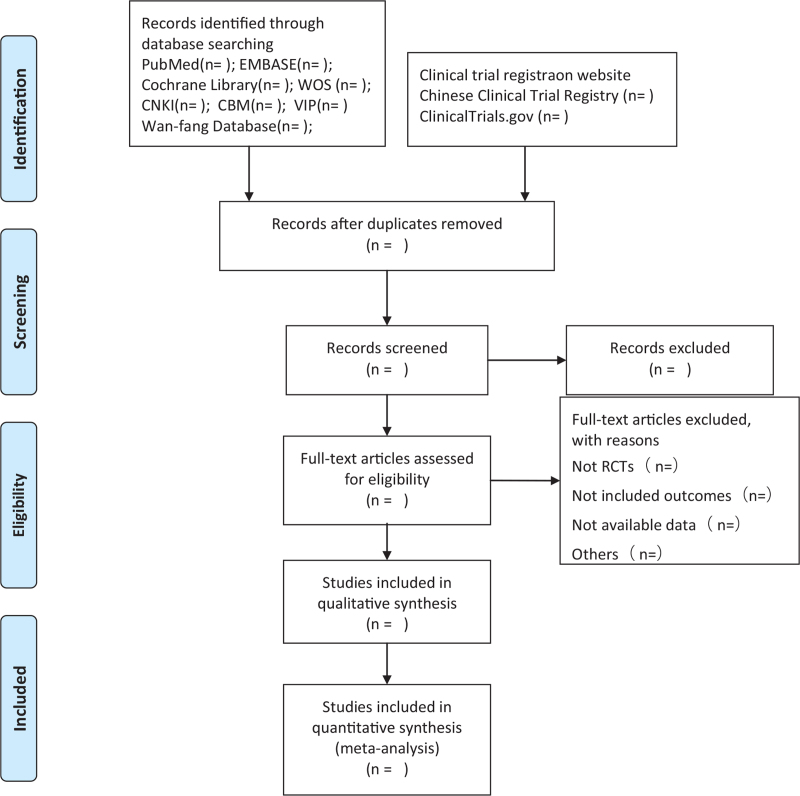
Flow diagram of study selection process.

First, the titles and abstracts of the studies will be independently reviewed by 2 review authors (ZHY and GHY), with preliminary screening of potential leads based on our predetermined eligibility criteria. Second, the full texts of all preliminary selective tests will be downloaded to ensure that the eligible tests are met. Unclear or missing information will be supplemented by contacting the authors. If there is any disagreement, a third reviewer (DTW) will participate in the consultation to reach consensus. The preferred reporting items for systematic reviews and meta-analyses (PRISMA) flow chart (Fig. 1) will be used to describe the selection process.

#### 2.5.2 Data extraction and management

The authors (ZHY and GHY) plan to extract the data from the articles selected for inclusion, and to resolve differences in opinion through discussion with experts. Data will be recorded on an electronic form, which includes identification information (author, working location, publication date, publication source, etc), patients (course, sex, age, number), intervention and control (method of intervention, method of control, sample size, and treatment time), and outcomes (treatment efficacy, treatment cycles, side effects, and follow-up), and so on.

#### 2.5.3 Assessment of risk of bias in included studies

The assessment will be conducted by 2 reviewers (ZHY and GHY) using the risk-of-bias assessment method from Cochrane Reviewer's Handbook 5.0.24.^[18]^ The main content comprised 7 items: random sequence generation, allocation concealment, blinding of participants and personnel, blinding of outcome assessment, incomplete outcome data, selective reporting, and bias. “High risk”, “low risk”, or “unclear risk” will be used to determine the result above. Disparities will be resolved by discussion and consultation with other authors in our group, and then a judgment will be made based on consensus.

#### 2.5.4 Measures of treatment effects

Two authors will perform the analysis independently and then crosscheck the treatment effect with Review Manager version 5.3 software. The risk ratio (RR) with a 95% confidence interval (95% CI) will be used for dichotomous data. The standard mean difference (SMD) or standard deviation (MD) with a 95% confidence interval will present continuous results. Other binary data are converted to the RR form.

#### 2.5.5 Dealing with missing data

To a certain extent, missing data had an impact on the results of this study. Therefore, for studies with missing data, we will contact the corresponding author and request further examination of missing or incomplete data in the record. If relevant data were not available or missing data were not available, we excluded them from the analysis.

#### 2.5.6 Assessment of heterogeneity

The research will be performed using the Review Manager version 5.3 software. Differences were considered statistically significant at *P* < .05. The *I*^2^ statistic was used to assess the level of heterogeneity of each pairwise comparison. If *I*^2^ < 50%, the fixed-effects model will be used; otherwise, a random-effects model will be used. When the results show substantial heterogeneity or considerable heterogeneity, sensitivity analysis or meta-regression and subgroup analysis will be performed to explore possible sources.

#### 2.5.7 Assessment of reporting bias

Publication and other reporting biases were assessed by creating funnel plots. The symmetrical funnel plots showed a low bias risk, whereas the asymmetric funnel plots showed a high bias risk.

#### 2.5.8 Data synthesis

We will also consider whether to conduct a meta-analysis based on clinical research. Meta-analysis was conducted according to the intervention methods, measurement methods, and heterogeneity levels of the clinical research. If the clinical and methodological heterogeneity is low, the fixed-effect model of combined analysis is adopted; when the heterogeneity is at a medium level, the combined analysis adopts a random-effect model.

### 2.6 Sensitivity analysis

We conducted a sensitivity analysis to test the robustness of key decisions made in the evaluation process. The central decision node mainly includes the selection of method quality, sample size, and missing data, and observes the fluctuation of the results.

### 2.7 Subgroup analysis

If the above clinical trials lead to significant heterogeneity, we will conduct a subgroup analysis according to the characteristics (interventions, different controls, treatment time, and outcome measurements) of other studies. We tabulated the sources of heterogeneity and then evaluated and explored them.

## 3 Discussion

PD is a common female disease, and severe cases will bring inconvenience to patients’ lives and work and even bring mental pressure and economic burden.^[19]^ External TCM treatment includes many effective techniques, such as acupuncture, moxibustion, massage, ear acupuncture, and acupoint application. The application of TCM external therapy for PD has been widely accepted in China, and has achieved remarkable results. However, due to the lack of direct comparisons between different TCM external treatments, clinicians are unable to choose the best treatment method.^[20,21]^ Clinicians usually choose 1 or several external therapies based on their experience to determine the most suitable treatment plan for patients. This situation increases the time and cost of treatment for patients and wastes the medical resources. Therefore, this study aimed to summarize the evidence from RCTs of various external TCM therapies for PD. To explore the effectiveness, safety, and advantages of all kinds of TCM external therapies in the treatment of PD and to provide clinicians and patients with the best treatment of the disease.

There are also some limitations to this study, such as the low quality of the original study, different intervention methods, and language limitations. This may have caused bias to a certain extent. Despite these limitations, this study will not only help establish a better way to prevent and treat patients with PD but also potentially improve the quality of life of patients with PD worldwide.

## Author contributions

Haiyu Zhu and Hongyi Guan had the original idea of this work and drafted the protocol. The search strategy was developed by all authors and will be performed by Haiyu Zhu, Hongyi Guan, Tingwei Ding, Yunpeng Bi, Yuanyuan Chen et al. Zhihong Wang proposed advice for design and revision. Yunpeng Bi and Tingwei Ding independently collected and extracted eligible studies. Yuanyuan Chen and Yue Zhuo assessed the bias risk and dealt with missing data. All the authors who participated in this study critically revised the final version of the manuscript and confirmed the publication of this protocol.

**Conceptualization:** Haiyu Zhu, Hongyi Guan.

**Data curation:** Tingwei Ding, Yunpeng Bi.

**Formal analysis:** Yuanyuan Chen.

**Funding acquisition:** Zhihong Wang.

**Investigation:** Haiyu Zhu, Tie Li.

**Methodology:** Haiyu Zhu, Hongyi Guan.

**Supervision:** Zhihong Wang.

**Validation:** Yue Zhuo, Tie Li.

**Writing – original draft:** Haiyu Zhu.

**Writing – review & editing:** Haiyu Zhu, Hongyi Guan, Zhihong Wang.
